# Phylogenetics Applied to Genotype/Phenotype Association and Selection Analyses with Sequence Data from Angptl4 in Humans

**DOI:** 10.3390/ijms11010370

**Published:** 2010-01-25

**Authors:** Taylor J. Maxwell, Matthew L. Bendall, Jeffrey Staples, Todd Jarvis, Keith A. Crandall

**Affiliations:** 1 Human Genetics Center, University of Texas School of Public Health, Houston, TX 77030, USA; 2 Department of Biology, Brigham Young University, Provo, UT 84602, USA; E-Mails: matthew.bendall@gmail.com (M.L.B.); grapas2@gmail.com (J.S.); todd.jarvis@gmail.com (T.J.); keith_crandall@byu.edu (K.A.C.)

**Keywords:** ANGPTL4, TreeSAAP, treescan, phylogenetics, association studies, selection

## Abstract

Genotype/phenotype association analyses (Treescan) with plasma lipid levels and functional site prediction methods (TreeSAAP and PolyPhen) were performed using sequence data for ANGPTL4 from 3,551 patients in the Dallas Heart Study. Biological assays of rare variants in phenotypic tails and results from a Treescan analysis were used as “known” variants to assess the site prediction abilities of PolyPhen and TreeSAAP. The E40K variant in European Americans and the R278Q variant in African Americans were significantly associated with multiple lipid phenotypes. Combining TreeSAAP and PolyPhen performed well to predict “known” functional variants while reducing noise from false positives.

## 1. Introduction

No single method of analysis is sufficient to uncover all the information that can come from sequence data. What we can strive for is a set of methods that complement each other. For example, the fields of molecular evolution, phylogenetics, and population genetics have a long history of sequence analysis [1,2]; however these methods do not typically use phenotype information. Many of these methods use knowledge about gene structure, amino acids, protein structure, and phylogenetics. We can borrow methods from these fields to identify polymorphic sites that may show evidence for selection or are likely to cause significant changes in expression or the nature of a protein.

Romeo *et al*. [3,4] sequenced the exonic regions and boundaries for the ANGPTL4 (angiopoietin-like protein 4) gene in patients from the Dallas Heart Study [5]. Results from analysis of these data [3] and subsequently in other ANGPTL genes [4] found that rare variants substantially contribute to variation in triglyceride levels. These groundbreaking papers substantiated these claims with biological assays showing that most rare variants in individuals in the tails of the triglyceride phenotypic distribution were functionally important by affecting secretion, expression, LDL inhibition, or loss of function. These results and data give us a rare opportunity to use “known” functional variants to assess the relative abilities of some site prediction methods such as PolyPhen [6] and TreeSAAP [7].

Using this data, we performed a series of analyses using phylogenetic approaches. We used Treescanning [8] to identify variants associated with lipid phenotypes. We used PAML [9] and HyPhy [10] to describe selection patterns across the sequence. Finally, we used the known rare functional variants from Romeo *et al.* [3,4] and results for common variants from the Treescanning analyses to compare the relative specificity and sensitivity of PolyPhen, TreeSAAP, and various combinations of the two.

## 2. Results and Discussion

### 2.1. Phylogenetic and Treescanning Results

#### 2.1.1. Variants, Haplotypes, Networks, and Phylogenetic Trees

Including a human reference sequence, there were 39 variants (27 missense, 11 synonymous) that produced 45 unique haplotypes. One missense mutation (G77R) from the previous study [3,4] was not included because the individual that harbored it had too much missing data to reliably infer its haplotypes. Four other variants (IVS3+1, K217X, FsK245, and FsS302) that were nonsense, frame shifts, or splicing mutations were not included in the selection analyses because the selection detection methods only consider amino acid replacements.

The haplotype inferences were relatively easy because most individuals were heterozygous for only one site. Technically, the singleton variants cannot be definitively placed on a haplotype unless it is heterozygous for only that site. However, because Treescanning uses genotypes, these individuals will always be grouped in the heterozygous class when the two possible haplotype backgrounds are defined as different allele classes making the test invariant to the phasing of the singleton. Regardless, the treescanning results were the same when all singletons were excluded. As for the phylogenetic analyses, the short branch lengths suggest that they will have little impact on analysis. For TreeSAAP, the same substitution event will always be inferred as long they are seen as tips.

**Figure 1.**
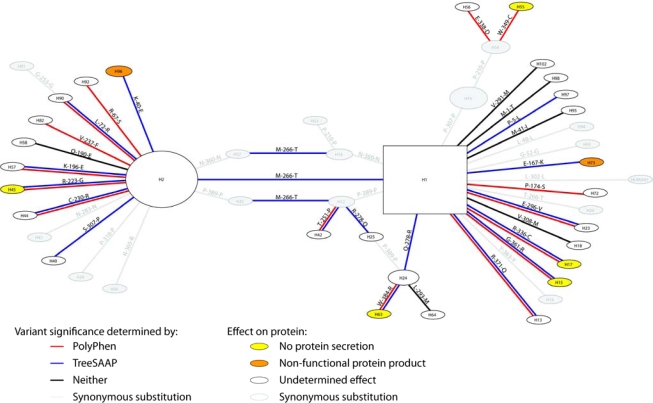
Phylogenetic network showing relationships among sampled haplotypes. Edges are labeled with the base or amino acid change and colored based on results from significant.PolyPhen and TreeSAAP results. The nodes are colored according to the known effect of the variant on the protein as determined by *in vitro* assays [3,4]. Yellow nodes indicate variants that prevent secretion; orange nodes indicate variants that cause a nonfunctional protein to be secreted; white nodes were not tested *in vitro*; and gray is a synonymous substitution.

The bootstrap analysis for the maximum likelihood (ML) tree revealed low resolution throughout the tree. The reason for such low resolution is the shortness of the branches. The haplotype network (Figure 1) illustrates this. Every single branch in the tree is only one step long, meaning that no haplotype is more than one site different than its nearest neighbor in the network. Bootstrapping works by sampling sites with replacement, which means that a site on a particular branch will be excluded in some of the replicates. Only branches with many sites will show any confidence in a bootstrap analysis. However, in coalescent theory, these short connections are considered more likely. Statistical Parsimony [11] was designed to incorporate these criteria when the haplotypes are sampled within a population. Another feature of this network is that two haploypes (H1 and H2) represent 70% to 80% of each population (see Table 1). Almost every other haplotype is a single step from either of these two haplotypes.

#### 2.1.2. Treescanning Results

As found by Romeo *et al.* [3], the branch carrying the E40K variant in the European American population was associated with various phenotypes. It was significant after correcting for multiple tests for triglycerides (multiple p = 0.0277), LDL (multiple p = 0.0141), and nominally for VLDL (nominal p = 0.0147, multiple p = 0.065). The full multivariate model for E40K is significant after multiple tests (multiple p = 0.0064). The univariate p-values are significant for triglycerides, LDL, and VLDL; however, the partial Wilk’s tests are only significant for LDL (p = 0.001) and nearly for triglycerides (p = 0.0556). This suggests that LDL probably contributes most to the association in the presence of the other variables followed by triglycerides while the univariate association of VLDL is probably accounted for correlations between the phenotypes. E40K was also nominally significant for triglycerides in the Mexican American populations even with a very small count of nine heterozygotes carrying the K variant (haplotype H96). It displayed the same protective effect of lower triglyceride levels as that found in the European Americans. No other variants within the European American population were significant in the second round of Treescanning.

The branch carrying the R278Q variant was significant for HDL (multiple p = 0.0122) in the African American population. The Q allele (carried by haplotypes H23, H24, H63, and H64) is fairly common in African Americans (see Table 1) at about 5.8% but is very rare in all other populations. The full multivariate model was not significant after multiple test corrections but was nominally significant (nominal p = 0.0394). Triglycerides were nominally significant for the univariate test (nominal p = 0.0359) but this effect went away using the multivariate context of a partial Wilk’s test (p = 0.3814); however, HDL-c remained significant using the partial Wilk’s test (p = 0.0098). The QQ homozygote shows much higher adjusted HDL-c levels (67.88 mg/dL; n = 7) *versus* the RQ heterozygote (55.13 mg/dL; n = 156) and RR homozygote (52.06 mg/dL; n = 1263). This finding has not previously been reported, however the sample of the QQ homozygotes is relatively small (n = 7). The significance of R278Q still held up, even after conditioning for E40K in the African American population (multiple p = 0.0108).

Talmud *et al*. [12] found mild evidence for an association with triglycerides with the T266M variant which is also the variant that separates the two major haplotypes (H1 and H2) in the network (see Figure 1). They found that this effect went away after conditioning upon the E40K variant. It is easy to see in Figure 1 how this variant could show an effect due to correlation with the E40K variant. Historically, the 40K mutation occurred on a 266M background resulting in linkage disequilibrium (LD) between the two variants. However, Talmud *et al.* [12] also found that T266M (but not E40K) was associated with postprandial triglyceride and glucose levels in a case/control study for individuals with a paternal history of myocardial infarction. In our study, T266M shows no association with any of the phenotypes and in any of our populations. At present, we do not have any data on postprandial stress to follow up their significant association with T266M.

### 2.2. Bioinformatics and Site Prediction Analysis Results

#### 2.2.1. PolyPhen and TreeSAAP Results

PolyPhen identified eight residues as “probably damaging” and seven as “possibly damaging” for a total of 15, leaving 12 as benign (see Table 2). Of the eight residues that were either functional or significant, PolyPhen identified five as “probably damaging” and three as benign. TreeSAAP identified 10 at category 8, 5 with category 6 or 7, and 12 as nothing. Of the eight residues that were either functional or significant, TreeSAAP identified five at category 8, two with category 6 or 7, and one as nothing. While both methods predicted similar numbers of sites under selection, only nine sites were found in common between the methods while 10 sites were unique to a particular method.

#### 2.2.2. PAML and HyPhy results

The PAML [9] M8 site prediction analysis did not find any sites under positive selection. A likelihood ratio test between the null model (M7) and the positive selection model (M8) did not support a class of sites under positive selection (ω > 1). The dual-rate random effects analysis implemented in HyPhy [10] did not detect positive selection at the absolute threshold of 0.95. Purifying selection was only detected on sites where synonymous substitutions had occurred. We conclude that likelihood-based site prediction methods were ineffective at identifying functional variants for our data. Our findings correspond to other studies concluding that TreeSAAP is more sensitive than likelihood-based site prediction methods for identifying sites under adaptive selection [13,14].

**Figure 2.**
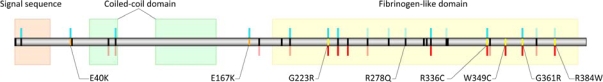
A schematic representation of ANGPTL4 coding region. The locations of variant sites are colored according to their affect on protein functionality as previously described [4]. Yellow sites prevent protein secretion, orange sites cause a non-functional protein to be secreted, and black sites were not tested *in vitro*. Amino acid sites identified by PolyPhen as “possibly damaging” are indicated in light red; “probably damaging” sites are shown in dark red. Radically changing (categories 6, 7, and 8) amino acid sites identified by TreeSAAP are shown in blue, with category 8 sites in dark blue.

However, likelihood-based methods proved to be useful in characterizing the selective constraint over distinct functional regions of ANGPTL4. We used the one ratio method (M0) implemented in PAML [9] to estimate the variation in functional constraint across the protein. The value of ω for the coiled-coil and fibrinogen-like domains is 1.057 and 0.386, respectively (see Figure 2). Using ω = 1 as the threshold between positive and negative selection, these results indicate that the coiled-coil domain is under nearly neutral selection, while the fibrinogen-like domain is under strong purifying selection. However, the metric of ω = 1 has been shown to underestimate the true amount of selective pressure on a protein region [15]. We estimated the value of ω to be 0.480 for the entire coding region. By using ω = 0.480 as a baseline, the coiled-coil domain appears to be under positive selection with respect to the rest of the gene, while the fibrinogen-like domain is under slightly negative (purifying) selection. These results suggest that the fibrinogen-like domains are under stronger functional constraint than the coiled-coil domain.

The two domains of ANGPTL4 each have unique selective pressures that are driving the evolution of these domains. Post-translational processing cleaves the coiled-coil and fibrinogen-like domains. The coiled-coil domain is involved in the inhibition of LPL, which results in high triglyceride levels. The exact function of the fibrinogen-like domain is not well known. However, it is clear that the functional role performed by each domain is vastly different, and these differences in function would imply a specific set of evolutionary constraints. This is affirmed by the discrepancy between nonsynonymous and synonymous substitution rate ratios. It is interesting to observe that the five variants found to affect secretion from *in vitro* assays are all found in the fibrinogen-like domain (Figure 2).

### 2.3. Comparison of PolyPhen and TreeSAAP

While both PolyPhen and TreeSAAP identified similar numbers of mutations under selection, they differed considerably in terms of which mutations each identified (Table 3). Only two criteria show a significant difference between the Functional column and the “Middle or Not Sig” column: The TreeSAAP alone criteria or the Strict PolyPhen and Strict TreeSAAP criteria. Both share a very high sensitivity (87.5%) however the TreeSAAP alone criterion has a slightly higher false positive rate (41.2%) and also misclassifies the two high-tail nonfunctional variants. As expected, there is a trend of lower false positive (alpha) rates as we move to the stricter criteria, which is also accompanied by lower sensitivity (power). The Strict PolyPhen & Strict TreeSAAP criteria for significance have the highest specificity but also the lowest sensitivity. The Strict PolyPhen criteria may have the best combination of specificity and sensitivity.

The purpose of these comparisons is to determine what is the best way to use these methods to define a subset of variants for biological assays and/or association analyses. For rare variants, individual association tests are meaningless; however, the phenotype data can be used in conjunction with these methods to narrow the likely candidates. In this case, most of the rare variants in the tails of triglyceride were functionally relevant according to biological assays. If these are a subset of variants sent for testing, both PolyPhen and TreeSAAP perform very well. TreeSAAP was able to identify five of the six rare functional low-tail functional variants plus the two phenotypically associated variants while wrongly finding the two high-tail nonfunctional variants as significant. The two misclassifications disappear when moving to more strict criteria where both methods are identical.

Both methods can be complementary because they give different information and have different aims. PolyPhen attempts to determine if a variant will damage a protein. TreeSAAP tries to identify mutations that are extremely out of the norm relative to the substitution patterns observed in the data for a specific biochemical property. The Strict PolyPhen and Strict TreeSAAP criteria suggest a variant has a high likelihood of importance by a least one method. In many cases, both methods give significant results because a variant is both damaging and it is a very extreme mutation according to the empirical data. It is not surprising that these two methods, which differ in their criteria for determining selection, differ in their outcomes. What is more surprising is that these methods that explore functional differences perform much better than the approaches (PAML and HyPhy) that simply look at dn/ds ratios. Clearly with these population genetic data, examining functional differences seems to provide greater insights into sites under natural selection.

Based on these limited results, we recommend a combination of the two methods that look at functional variants in a population to be most desirable for choosing variants to create *a priori* tests. If the investment in following up with biological assays is very high then the Strict PolyPhen and Strict TreeSAAP criteria are a very strict filter that together have the lowest false positive rate. However, if the goal is to be inclusive, the Strict PolyPhen OR Strict TreeSAAP criterion was very sensitive while still lowering the false positive rate.

## 3. Materials and Methods

### 3.1. Study Description and Genetic Data

The Dallas Heart Study is based on a population sample restricted to the Dallas area [5]. That is, individuals in the sample were ascertained randomly without reference to their phenotypic values or disease status. The samples sequenced for the ANGPTL4 gene contains 3,551 individuals (1,830 African Americans, 601 Hispanics, 1045 European Americans, and 75 other ethnicities). All exons from each gene were sequenced along with each intron/exon boundary. All sequencing was done at the Joint Genome Institute. Base calling, quality assessment and assembly were carried out using the Phred, Phrap, Polyphred, Consed software suite. All sequence variants identified were verified by manual inspection of the chromatograms, and missense mutations were confirmed by independent resequencing [3,16]. Five quantitative lipid measures related to heart disease were analyzed: Triglyceride, HDL, VLDL, LDL, and total cholesterol levels.

### 3.2. Haplotype Networks and Phylogenetic Trees

All exonic regions were aligned and haplotypes were statistically inferred from the genotype data, using PHASE 2.2 [17,18]. A haplotypes network was inferred using a modified version of TCS [19]. The haplotype tree showed no evidence for recombination [20]. Coalescent criteria [21,22] allowed for resolution of each loop by breaking the H16-H37, H12-H35, and H24-H25 branches in Figure 1.

Likelihood scores were calculated from the sequences for the unique haplotypes for 56 models of nucleotide evolution using PAUP* [23]. We determined the best-fit model of nucleotide evolution using a maximum likelihood ratio test implemented in Modeltest [24]. The HKY model [25] with a gamma distribution shape parameter of 0.0104 and a ti/tv ratio of 2.1982 was determined to be the best model given the data. A phylogenetic tree was estimated using the maximum likelihood criterion as implemented in the application PhyML [26]. Branch support for the tree was estimated using non-parametric bootstrap sampling with 1,000 replicates. The ML tree was used for all analyses with TreeSAAP, PAML, and HyPhy.

### 3.3. Genotype/Phenotype Association via Treescan

After being adjusted for age, sex, and BMI, separate analyses for cholesterol, triglyceride, VLDL, LDL, and HDL levels were performed separately for African-Americans, Mexican-Americans, and European-Americans. Romeo *et al*. [3,4] found a number of rare variants that were functionally significant through biological assays. These known effects may group in ways that may affect associations at other common polymorphisms and branches in the network. Therefore, analyses were performed with and without the individuals harboring these variants. The estimated haplotype network was used for all Treescan [8,27,28] analyses. All treescanning analyses used genotypes as factors and only included genotypic classes with counts of five or more. All nominal and multiple-test corrected significance levels were obtained with 10,000 permutations. A permutation analog of the sequential step-down Bonferroni [29] was used for multiple test correction because it takes into account the correlation between tests.

Because the five lipid phenotypes are biologically related to each other through hepatic and intestinal lipid metabolism, the results from the univariate Treescan analyses were tested in a multivariate one-way MANOVA model where each branch is jointly associated with triglyceride, HDL, VLDL, and LDL levels. Total cholesterol is excluded because it is a composite value of the other three. Significance levels will be derived in a similar fashion using the parametric p-value from the F transformation of the Wilk’s statistic. A partial Wilk's test can be used to test the effects of individual dependent (phenotypes) or independent variables while controlling for all the other variables in the model. The partial Wilk's test is a reduced *versus* full model approach. This conditional Wilk's statistic is calculated as follows [30]:

(1) $$\Lambda (y_{g}|y_{1},\ldots ,y_{g-1},y_{g+1},\ldots ,y_{p})=\frac{\Lambda_{p}}{\Lambda_{p-1}}$$

where *p* is the number of phenotypes (dependent variables), y_g_ is the phenotype of interest, Λ*_p_* is the Wilk's statistic for the full model, and Λ_*p–*1_ is the Wilk's statistic for a reduced model where y_g_ is excluded. The resulting partial Wilk's statistic has an exact transformation to a partial F-statistic [30]:

(2) $$F_{v_{H},v_{E-p+1}}=\frac{1-\Lambda }{\Lambda }\frac{v_{E}-p+1}{v_{H}}$$

where Λ is the result of equation 1,*p* is the number of phenotypes, v_E_= N – k, v_H_= k – 1, N = number of individuals, and k = the number of factor levels in the one-way MANOVA. The partial Wilk's test measures the contribution of a single phenotype to the genotypic association in the presence of the other phenotypes across all eigenvectors of the **E** ^−1^**H** matrix. Univariate F tests and partial Wilk's tests are calculated for each significant MANOVA result emerging from the initial Treescan.

### 3.4. Bioinformatics, Site Prediction, and Selection Analyses

#### 3.4.1. PolyPhen Analysis

Some nonsynonymous variants are benign and have little to no effect on protein function, while others can be extremely harmful and cripple the protein. A variant’s effect on protein function can be predicted using a multiple protein alignment to assign each variant a score of how harmful the variant will be to protein function *via* the software PolyPhen [6] (e.g., a PolyPhen score of 0 = benign and a score of 4 = probably damaging). Given the 3D protein structure for ANGPTL4 is unknown, PolyPhen predictions were based predominantly on an alignment of the homologous sequences obtained through a Blast search of the NRDB database. The 45 unique SNPs were submitted to and retrieved from the PolyPhen web server using batch submission and retrieval scripts.

#### 3.4.2. TreeSAAP Analysis

Another approach to identifying sites that are subject to adaptive change is by analyzing the changes in physiochemical properties when a substitution occurs [31]. Substitutions are determined by reconstructing ancestral states given a phylogenetic tree. Operating under the assumption of completely random amino acid replacement, we can calculate the expected distribution of amino acid substitutions. The substitutions inferred from the ancestral states are then compared to the expected distribution to determine the significance of the observed changes *via* the software package TreeSAAP [7]. The ancestral character states used by TreeSAAP are estimated using BaseML, which is part of the PAML software package [9]. We analyzed 31 different physiochemical properties, with 8 magnitude categories. Substitutions with changes of magnitude 6, 7, and 8 are considered to be radically changing [13] and are used in this paper to indicate significant variants.

#### 3.4.3. Likelihood Selection Analysis

We used several likelihood-based methods to estimate the influence of selection on ANGPTL4. Likelihood methods use a codon-based model [32] of evolution to estimate the nonsynonymous to synonymous rate ratio (ω). A value of ω > 1 is commonly thought to be an indicator of positive selection, ω = 1 is neutral evolution, and ω < 1 indicates purifying selection. We implemented the M8 model in PAML [9], which allows the nonsynonymous rate to vary among sites, while the synonymous rate is assumed to be homogeneous. The dual-rate model, implemented in HyPhy [10], allows both the nonsynonymous and synonymous rates to vary between sites, which has been shown to have greater power when compared to models where only the nonsynonymous rate is allowed to vary [10]. Both methods were used to infer sites under selection across the entire coding sequence.

Maximum likelihood was also used to estimate overall levels of selection in each of the protein domains and across the entire coding sequence. The coiled-coil and fibrinogen-like domains were separated, and ω was computed independently for each region. We used the one ratio (M0) model implemented in PAML [9], which assumes that ω is constant across all sites.

### 3.5. Comparison of TreeSAAP and PolyPhen

Of the 27 nonsynonymous variants analyzed, the functional polymorphisms from the biological assays [3,4] and the significant variants from Treescan analyses will be treated as “known” functional variants from which to evaluate the results of TreeSAAP, PolyPhen and their combination. We will compare PolyPhen, Strict PolyPhen (only “probably damaging”), TreeSAAP, Strict TreeSAAP (only category 8), PolyPhen and TreeSAAP, and Strict PolyPhen and Strict TreeSAAP. The remaining variants of the 27 will be defined as nonfunctional. This is conservative because only variants in the tails of triglyceride were biologically tested. A two-tailed Fisher’s exact test was performed on a 2 by 2 table with the rows being the results of the method and the columns being the “known” information on the variants. If a method performs well, we would expect that it should have a higher ratio in the functional column than nonfunctional column. Biological assays were performed on eight variants (2 in the high tail and 6 in the low). All six low-tail variants were shown to have some type of functional effect. The two high-tail variants did not show any functional evidence. These two variants were classified separately from the other variants.

## 4. Conclusions

From our study, we had three different types of analyses: Genotype/phenotype association (Treescan), overall selection analyses (PAML M0), and three site prediction methods (REL, PolyPhen and TreeSAAP). Besides PolyPhen, each type of analysis used some form of phylogenetic data, and each gave us additional insight. First, the Treescan analysis provided evidence for an association with HDL in African Americans with the R278Q variant. Second, the PAML M0 analysis demonstrated the coiled domain is under positive selection while the fibrinogen-like domain is under slightly negative selection. It is of interest that most of the rare functional variants are within the fibrinogen-like domain. Finally, the “known” functional variants were leveraged such that we could evaluate the relative merits of site prediction from PolyPhen and TreeSAAP. We concluded that a combination of both methods is likely the best approach to take.

While no sequence analysis method is going to reveal everything about genotype/phenotype relations, we do have tools that can work together to give us greater insight and lead us towards productive paths. For association studies, sequence data can give us greater ability to estimate the phylogenetic relationships between haplotypes. This in turn leads to a greater context for which to direct and interpret statistical tests. For TreeSAAP, phylogenetic estimation allows for an empirical estimate of the distributions of different types of amino acid changes. From these distributions, we can make predictions about which particular changes are out of the ordinary and are more likely to have an impact on gene function and subsequently on the phenotypes that we are interested in.

In future studies, these site prediction methods will be a first step to provide greater statistical power and impetus to invest in biological follow up. These methods create *a priori* hypotheses to be tested leading to greater statistical power with the reduced number of tests to correct for. These methods may also suggest the biological nature of sites predicted to have functional consequence. Many labs are currently embarking on whole exome sequencing. These methods will be useful as we try to comb through this mass of data to separate the functional from nonfunctional variants.

## Figures and Tables

**Table 1.** Haplotype frequencies for the haplotypes in Figure 1 overall and each population. All = combined; EA = European American, AA = African American, MA = Mexican American.

**Haplotype**	**All**	**EA**	**AA**	**MA**	**Other**
h1	0.51242	0.54341	0.47735	0.54052	0.68493
h2	0.26020	0.28447	0.21706	0.34828	0.23288
h12	0.06475	0.14402	0.02102	0.05862	0.06164
h13	0.00015	0	0.00030	0	0
h14	0.00015	0.00051	0	0	0
h15	0.00030	0.00102	0	0	0
h16	0.04186	0.00153	0.07936	0.00517	0.00685
h17	0.00105	0.00255	0.00030	0.00086	0
h18	0.00045	0.00102	0	0.00086	0
h19	0.01220	0	0.02399	0	0
h23	0.00015	0.00051	0	0	0
h24	0.02997	0.00051	0.05774	0.00259	0
h25	0.00015	0	0.00030	0	0
h26	0.00030	0	0.00059	0	0
h35	0.00045	0	0.00089	0	0
h36	0.00015	0	0.00030	0	0
h37	0.00211	0	0.00415	0	0
h39	0.00015	0	0.00030	0	0
h40	0.00015	0.00051	0	0	0
h41	0.00015	0	0.00030	0	0
h42	0.00015	0.00051	0	0	0
h44	0.00015	0	0	0.00086	0
h45	0.00015	0.00051	0	0	0
h53	0.00030	0	0.00059	0	0
h54	0.05285	0.00204	0.10127	0.00431	0
h55	0.00015	0	0.00030	0	0
h56	0.00015	0	0.00030	0	0
h57	0.00015	0.00051	0	0	0
h58	0.00467	0.00153	0.00651	0.00517	0
h63	0.00015	0	0.00030	0	0
h64	0.00015	0	0.00030	0	0
h72	0.00015	0	0.00030	0	0
h73	0.00015	0.00051	0	0	0
h82	0.00015	0.00051	0	0	0
h90	0.00015	0	0.00030	0	0
h91	0.00030	0	0.00059	0	0
h92	0.00030	0.00051	0.00000	0	0.00685
h93	0.00060	0	0.00118	0	0
h94	0.00015	0	0.00030	0	0
h95	0.00407	0	0	0.02328	0
h96	0.00708	0.01277	0.00296	0.00948	0.00685
h97	0.00030	0	0.00059	0	0
h98	0.00015	0	0.00030	0	0
h102	0.00015	0.00051	0	0	0

**Table 2.** PolyPhen and TreeSAAP results for each missense polymorphisms used in the study. Each rare variant is defined by which part of the triglyceride phenotype distribution it was found (H = high, M = Middle, L= Low) according to Romeo *et al.* [3]. For the five common missense variants, Significant and NonSig (Nonsignificant) refers to phenotypic associations from the Treescan analyses. The Biological Assay column refers to assays in Table 3 of Romeo *et al*. [4]. A “-“ means no tests were performed. All significant PolyPhen predictions are in bold. All TreeSAAP properties considered significant with a score of 6 or more [13] are reported, all with an extreme value of 8 are in bold. The TreeSAAP property symbol key is provided below.

**Missense Variant**	**Phenotype Distribution**	**Biological Assay**	**PolyPhen Score**	**PolyPhen Prediction**	**TreeSAAP Property**
M-1-T	M	-	NA	benign	
P-5-L	M	-	NA	benign	**αc**, **αn**, K0, Hp
E-40-K	Significant	-	1.424	benign	**pHi**
M-41-I	NonSig	-	1.16	benign	
S-67-R	M	-	1.563	**possibly damaging**	
R-72-L	M	-	1.958	**possibly damaging**	**H**, **Hnc**, αn
E-167-K	L	LPL Inhib	0.194	benign	**pHi**
P-174-S	M	-	1.715	**possibly damaging**	
E-190-Q	NonSig	-	0.243	benign	
E-196-K	M	-	1.541	**possibly damaging**	**pHi**, El
G-223-R	L	Secretion	2.065	**probably damaging**	**E’sm**
R-230-C	M	-	2.792	**probably damaging**	**pHi**, **E’sm**, **Et**, **Br**, Ns, C
F-237-V	M	-	2.51	**probably damaging**	
P-251-T	H	Nothing	1.781	**possibly damaging**	
T-266-M	NonSig	-	0.783	benign	K0, Ht
R-278-Q	Significant	-	0.644	benign	pHi
V-291-M	M	-	1.012	benign	
L-293-M	M	-	1.236	benign	
E-296-V	M	-	2.057	**probably damaging**	**Ns**, **Pβ**, Br, H, Ra
P-307-S	M	-	0.955	benign	αc
V-308-M	M	-	1.199	benign	
R-336-C	L	Secretion	2.255	**probably damaging**	**Br**, **pHi**, **Et**, Ns, C, Ca, Hnc
D-338-E	M	-	1.626	**possibly damaging**	
W-349-C	L	Secretion	3.677	**probably damaging**	
					**Ca**, **E’sm**, Mv, Mw, Hnc, V0,
G-361-R	L	Secretion	2.274	**probably damaging**	μ
R-371-Q	H	Nothing	1.558	**possibly damaging**	pHi
R-384-W	L	Secretion	2.304	**probably damaging**	Br, Ht

**TreeSAAP Property Key**			
Alpha-helical tendency	Pα	Molecular weight	Mw
Average # of surrounding residues	Ns	Normalized hydrophobicity	Hnc
Beta-structure tendency	Pβ	Partial specific volume	V0
Buriedness	Br	Power to be at the C-terminal	αc
Composition	C	Power to be at the N-terminal	αn
Compressibility	K0	Refractive index	μ
Helical contact	Ca	Short-range & medium-range nonbonded energy	E’sm
Hydropathy	H	Solvent accessible reduction ratio	Ra
Isoelectric point	pHi	Surrounding hydrophobicity	Hp
Long-range nonbonded energy	El	Thermodyn. transfer hydrophobicity	Ht
Molecular volume	Mv	Total non-bonded energy	Et

**Table 3.** A comparison of results between PolyPhen, TreeSAAP, and their combinations with “known” data. Strict PolyPhen only counts “probably damaging” as significant while Strict TreeSAAP only counts category 8 as significant. P-values are from a two-tailed Fisher’s exact test of a 2 by 2 table comparing the “Functional or Significant column to the “Middle or Not Sig” column. Sensitivity, specificity, alpha, and beta levels are from this comparison.

**Significance Criteria**		**Functional or Significant**	**Tested Not Functional**	**Middle or Not Sig**	**p-val**	**Odds Ratio**	**Lower 95 CI**	**Upper 95 CI**
**PolyPhen**	Significant	5	2	8	0.673	1.828	0.254	15.766
				
	Not Significant	3	0	9				
				
	**Sensitivity**	0.625	**Specificity**	0.529	**alpha**	0.471	**beta**	0.375

**TreeSAAP**								493.08
	Significant	7	2	7	**0.042**	9.130	0.859	8
				
	Not Significant	1	0	10				
				
	**Sensitivity**	0.875	**Specificity**	0.588	**alpha**	0.412	**beta**	0.125

**Strict PolyPhen**	Significant	5	0	3	0.061	7.012	0.846	77.356
				
	Not Significant	3	2	14				
				
	**Sensitivity**	0.625	**Specificity**	0.824	**alpha**	0.176	**beta**	0.375

**Strict TreeSAAP**	Significant	5	0	5	0.194	3.762	0.505	34.675
				
	Not Significant	3	2	12				
				
	**Sensitivity**	0.625	**Specificity**	0.706	**alpha**	0.294	**beta**	0.375

**PolyPhen & TreeSAAP**	Significant	4	0	4	0.359	3.084	0.385	27.020
				
	Not Significant	4	2	13				
				
	**Sensitivity**	0.5	**Specificity**	0.765	**alpha**	0.235	**beta**	0.5

**Strict PolyPhen & Strict TreeSAAP**	Significant	3	0	2	0.283	4.192	0.369	64.438
				
	Not Significant	5	2	15				
				
	**Sensitivity**	0.375	**Specificity**	0.882	**alpha**	0.118	**beta**	0.625

**Strict PolyPhen OR Strict TreeSAAP**						11.52		626.87
	Significant	7	0	6	**0.030**	6	1.077	1
				
	Not Significant	1	2	11				
				
	**Sensitivity**	0.875	**Specificity**	0.647	**alpha**	0.353	**beta**	0.125
